# Associations between long-term exposures to environmental factors and the development of amyotrophic lateral sclerosis: A matched case-control study 

**DOI:** 10.23889/ijpds.v9i5.2790

**Published:** 2024-09-18

**Authors:** Daniel Saucier, Mathieu Bélanger, Zikuan Liu, Eric Lavigne, Colleen O'Connell

**Affiliations:** 1 Université de Sherbrooke; 2 Centre de formation médicale du Nouveau-Brunswick; 3 New Brunswick Institute for Research, Data and Training, University of New Brunswick; 4 Environmental Health Science and Research Bureau, Health Canada; 5 University of Ottawa; 6 Stan Cassidy Centre for Rehabilitation

## Objective

Amyotrophic lateral sclerosis (ALS) is a motor neuron disease in which the causes remain unclear, particularly the contribution of environmental factors. Thus, we created a multifactorial database to investigate the association between long-term exposure to urbanization, air pollution, water pollution, and the development of ALS.

## Approach

A matched case-control study was conducted in New Brunswick, Canada from January 2003 to February 2021. Study population included 304 ALS patients and 1207 controls with their historical postal codes from the New Brunswick Citizen Database linked to medical records and spatial environmental datasets from the Canadian Urban Environmental Heath Research Consortium. We compared their environmental exposures at place of residence prior to disease onset via conditional logistic regression models.

## Results

Of the common air pollutants investigated, odds of ALS was significantly higher with increased SO2 exposure (OR = 4.369, 1.190-16.044 [95% CI], per 1 ppb increase of SO2) in adjusted models. No significant associations were observed for the investigated urbanization and water pollution-related exposures.

## Conclusions

This is the first study to rigorously identify a potential environmental cause of ALS. Our findings suggest an association between long-term exposure to air pollutants, particularly SO2, and the development of ALS. Revision of SO2 emission regulations may be required.

## Implications

The identification of SO2 exposure as an ALS environmental risk factor opens new prospects for identifying ALS therapeutic targets. Utilizing a similar data linking workflow to pre-existing disease registries found within data centres should be conducted to further investigate environmental influences on complex diseases.

